# *Mycobacterium* Biofilms

**DOI:** 10.3389/fmicb.2017.02651

**Published:** 2018-01-18

**Authors:** Jaime Esteban, Marta García-Coca

**Affiliations:** Department of Clinical Microbiology, Instituto de Investigación Sanitaria-Fundación Jiménez Díaz, Madrid, Spain

**Keywords:** *Mycobacterium*, biofilms, antimicrobial resistance, *in vitro* study, review, rapidly growing mycobacteria, *Mycobacterium tuberculosis*, *Mycobacterium avium* complex

## Abstract

The genus *Mycobacterium* includes human pathogens (*Mycobacterium tuberculosis* and *Mycobacterium leprae*) and environmental organisms known as non-tuberculous mycobacteria (NTM) that, when associated with biomaterials and chronic disease, can cause human infections. A common pathogenic factor of mycobacteria is the formation of biofilms. Various molecules are involved in this process, including glycopeptidolipids, shorter-chain mycolic acids, and GroEL1 chaperone. Nutrients, ions, and carbon sources influence bacterial behavior and have a regulatory role in biofilm formation. The ultrastructure of mycobacterial biofilms can be studied by confocal laser scanning microscopy, a technique that reveals different phenotypic characteristics. Cording is associated with NTM pathogenicity, and is also considered an important property of *M. tuberculosis* strains. Mycobacterial biofilms are more resistant to environmental aggressions and disinfectants than the planktonic form. Biofilm-forming mycobacteria have been reported in many environmental studies, especially in water systems. NTM cause respiratory disease in patients with underlying diseases, such as old tuberculosis scars, bronchiectasis, and cystic fibrosis. Pathogens can be either slowly growing mycobacteria, such as *Mycobacterium avium* complex, or rapidly growing species, such as *Mycobacterium abscessus*. Another important biofilm-related group of infections are those associated with biomaterials, and in this setting the most frequently isolated organisms are rapidly growing mycobacteria. *M. tuberculosis* can develop a biofilm which plays a role in the process of casseous necrosis and cavity formation in lung tissue. *M. tuberculosis* also develops biofilms on clinical biomaterials. Biofilm development is an important factor for antimicrobial resistance, as it affords protection against antibiotics that are normally active against the same bacteria in the planktonic state. This antibiotic resistance of biofilm-forming microorganisms may result in treatment failure, and biofilms have to be physically eradicated to resolve the infection. New strategies with potential antibiofilm molecules that improve treatment efficacy have been developed. A novel antibiofilm approach focuses on *Methylobacterium* sp. An understanding of biofilm is essential for the appropriate management of patients with many NTM diseases, while the recent discovery of *M. tuberculosis* biofilms opens a new research field.

## Introduction

The genus *Mycobacterium* currently includes more than 170 species (Tortoli, 2006, 2014). Most of these species are environmental organisms that have never been implicated in human infection, whereas others are among the oldest human pathogens ever described. *Mycobacterium tuberculosis* and members of the *M. tuberculosis* complex are still among the most important causes of disease, and the epidemiological and social importance of *Mycobacterium leprae* is beyond doubt (Esteban and Muñoz-Egea, 2016). However, all other human pathogens are also environmental organisms that can be found in many different ecosystems without public health implications. These organisms, known as non-tuberculosis mycobacteria (NTM), can cause human infections in special circumstances, in many cases involving the presence of biomaterials; in other cases, they cause chronic infections in patients with underlying diseases or even outbreaks associated with environmental sources (Esteban et al., 2012). In this review, we will explore the importance of biofilms in mycobacterial disease and in environmental sources, and the implications of these structures in the diagnosis and treatment of mycobacterial diseases.

## History

The first report of the modern concept of biofilm dates from 1978, when Costerton et al. published their initial observations (Costerton et al., 1978). Another decade later, articles began to appear on environmental mycobacterial biofilms (Wallace, 1987; Schulze-Robbecke and Fischeder, 1989), even though the phenomenon of mycobacterial cells forming “aggregates” or “pellicles” was described in the early days of mycobacteriology (Löwenstein, 1920; Calmette, 1936): in a pivotal article on the etiology of tuberculosis, Robert Koch described the appearance of “cells which are pressed together and arranged in bundles” (Koch, 1982). Subsequent studies described *M. tuberculosis* forming “pellicles” in liquid media, with images (Calmette, 1936) quite similar to what in modern times are described as biofilms (Ojha et al., 2008). Similar descriptions for avian bacilli and others were also reported (Löwenstein, 1920), so it was clearly demonstrated that mycobacteria naturally grow in biofilm structures. Decades more then passed before laboratory methods were developed to achieve dispersed mycobacterial cell growth (Dubos and Davis, 1946; Pierce et al., 1947). Despite all this knowledge, however, it was not until the 1990s that the current concept of biofilm emerged from the first findings of modern research on mycobacterial biofilms.

## Characteristics of mycobacterial biofilms

Biofilms formed by mycobacteria can be defined in the same way as any other biofilms. However, some mycobacteria can develop these structures not only on surfaces, but also on the air-media interface (Ojha et al., 2008). This phenomenon may be explained by the different composition of the extracellular matrix of the biofilm and the unique characteristics of mycobacterial cell wall, especially the presence of high lipid levels. As with many other organisms, biofilm development starts with bacterial adhesion and then proceeds through the different stages of surface attachment, sessile growth, matrix synthesis, and dispersal. Intercellular communication occurs through a quorum-sensing phenomenon (Richards and Ojha, 2014). Different molecules from the bacterial cell wall called adhesins mediate the initial attachment of bacteria to the surfaces. Once attached to the surface, sessile bacteria initiate the synthesis of an extracellular matrix, usually composed of glycopeptides, DNA, and other molecules. However, mycobacteria lack surface fimbriae or pili, although certain proteins have been described as potential factors for the aggregation of mycobacteria and attachment to other cells (Menozzi et al., 1996). Nor do mycobacteria produce the usual exopolysaccharide components of extracellular matrix, but they can attach to different surfaces (Zamora et al., 2007) and form fully developed biofilms (Zambrano and Kolter, 2005; Ojha et al., 2015).

Several studies confirm that NTM have the ability to adhere to biomaterials. Vess et al. showed how several species of mycobacteria can adhere to polyvinyl chloride (Vess et al., 1993); Ridgway et al. analyzed the adherence of *Mycobacterium* sp. to the cellulose diacetate (Ridgway et al., 1984); and Zamora et al. studied the adherence of NTM to polypropylene (Zamora et al., 2007). In this last study, differences in adherence were verified, not only between species, but also between strains of the same species. After adherence, mycobacterial biofilm is formed. Another study of different species of rapidly growing mycobacteria (RGM) showed that biofilm development by these species follows a sigmoid growth kinetic (Esteban et al., 2008). This study in laboratory strains was later confirmed in clinical strains, and differences were noted depending on their clinical significance (Martin-de-Hijas et al., 2009). Nutrients, ions (Ca^2+^, Mg^2+^, and Zn^2+^), and carbon sources, such as glucose and peptone, are known to influence bacterial behavior and have a regulatory role in the formation of biofilm (Carter et al., 2003). Esteban et al. (2008) showed that RGM can develop biofilm using only tap water as the nutrient source, which may explain the detection of mycobacteria from water sources. Although *Mycobacterium avium* complex organisms were among the first mycobacterial biofilms ever described (Carter et al., 2003), many NTM have been found in heterospecies biofilms from environmental specimens (Falkinham, 2002, 2009).

Several studies have examined the role of different molecules in the formation of these biofilms and in their composition. Recht et al. showed that *Mycobacterium smegmatis* glycopeptidolipids are essential for initial surface attachment during biofilm formation (Recht and Kolter, 2001). These molecules also have an essential role in sliding motility, a property of many strains of mycobacteria that can be related with biofilm spreading to contiguous surfaces (Recht et al., 2000; Recht and Kolter, 2001; Maya-Hoyos et al., 2015). However, biofilm development and sliding motility are not always associated, according to another study in a large sample of RGM clinical strains (Martin-de-Hijas et al., 2009). Ojha et al. demonstrated that in non-motile mycobacteria, shorter-chain mycolic acids have important role in the development of biofilm structure (Ojha et al., 2008). They also proposed that these shorter mycolic acids may form a hydrophobic extracellular matrix (Ojha et al., 2005). The high resistance to antibiotics and disinfectants associated with these organisms is attributed mainly to mycolic acids, which help provide a permeability barrier (Zambrano and Kolter, 2005). Many other molecules have also been studied (Ojha et al., 2015). Ojha et al. explored the role of GroEL1 chaperone in the development of *M. smegmatis* biofilms (Ojha et al., 2005; Esteban et al., 2008), and the complexity of mycobacterial biofilm structure and development is also being investigated.

The ultrastructure of mycobacterial biofilms has also been studied with different methodologies. Confocal laser scanning microscopy (CLSM) combined with two fluorescent dyes, Nile Red^©^ (Sigma-Aldrich Co., St. Louis, MO, USA) and LIVE/DEAD BacLight^©^ (Invitrogen, USA), has been used to analyze the phenotypic characteristics of biofilms formed by some RGM (growth rate, percentage of covered surface, percentage of live/dead bacteria, and autofluorescence), showing differences between species (Muñoz-Egea et al., 2013). In this study, Muñoz-Egea et al. showed that maximum thickness for *Mycobacterium fortuitum* and *Mycobacterium chelonae* biofilm was detected at 72 h, but other non-pigmented RGM reach maximum thickness at 96 h. *M. chelonae* covered a smaller surface area than *Mycobacterium abscessus*, but a greater area than *M. fortuitum* and *Mycobacterium mageritense* (Muñoz-Egea et al., 2013). Interestingly, autofluorescence, which has been found among different mycobacterial species (Patino et al., 2008), can be detected not only in sessile bacteria but also in the extracellular matrix. Patino et al. speculate that coenzyme F420 could be involved in this phenomenon. This molecule may be secreted by the bacterial components of the biofilm, and is thus detectable in extracellular matrix using CLSM (Patino et al., 2008; Joshi et al., 2013; Muñoz-Egea et al., 2013).

Growth characteristics also differ between *Mycobacterium* species. *M. chelonae* forms a biofilm that grows vertically, while *M. fortuitum* covers the entire surface with a thinner growth. Extensive cording is observed in the cases of *M. abscessus* and *M. chelonae* (Muñoz-Egea et al., 2013). Cording is associated with pathogenicity (Williams et al., 2009; Muñoz-Egea et al., 2013), and the deletion of a dehydratase gene that affects cording made *M. abscessus* strains avirulent (Halloum et al., 2016). Cording has long been considered an important property of *M. tuberculosis* strains, and contributes to the pathogenesis of tuberculosis (Maya-Hoyos et al., 2015). The implications of cording and biofilm development by *M. tuberculosis* are still being investigated (Caceres et al., 2013).

## Mycobacterial biofilms in the environment

The ability of mycobacteria to live in biofilms confers these organisms many advantages over the planktonic form of growth, including, for example, resistance to environmental aggressions, and mycobacteria that grow in biofilms appear to be related to environmental sources (Schulze-Robbecke and Fischeder, 1989; Schulze-Robbecke et al., 1992; Schulze-Robbecke, 1993). Many reports of environmental studies have demonstrated the presence of these mycobacteria, especially in water systems (Falkinham, 2002, 2009). Other studies have shown the role of these reservoirs in the development of outbreaks caused by these organisms, including infections secondary to cosmetic interventions, medical procedures, and others (Meyers et al., 2002; Winthrop et al., 2002; Vijayaraghavan et al., 2006; van Ingen et al., 2009; Kennedy et al., 2012; El Helou et al., 2013b; Walker et al., 2017). Recently, *Mycobacterium chimaera* has emerged as an important nosocomial pathogen associated with contamination of heater-cooler units for cardiac surgery throughout the world (Kohler et al., 2015), and special decontamination measures have been necessary to remove mycobacterial biofilms from these devices (Garvey et al., 2016, 2017). In this respect, it is important to take into account that sessile mycobacteria show greater resistance to disinfectants than planktonic species. One report even described NTM detected in the bottom of a glutaraldehyde solution tray (Vijayaraghavan et al., 2006).

It has recently postulated that rising numbers of NTM isolated from clinical samples could be related to changes in disinfectants used in plumbing systems, in particular, the switch from chlorine to chloramine in water systems in the USA (Falkinham, 2016). Unexplained changes in the numbers of isolates have also been described (Esteban et al., 2007), so it seems likely that many questions regarding the ecology of these organisms remain to be clarified.

An interesting issue is the relationship between NTM and free-living amoebae (Vaerewijck et al., 2005). It is well-known that mycobacteria can be intracellular parasites, which may explain the potential relationship between NTM and amoeba in water and biofilm samples (Marciano-Cabral et al., 2010; Ovrutsky et al., 2013). These mixed eukaryotic-prokaryotic biofilms can be a source of human infections and a growing factor in resistance against disinfectants.

## Mycobacterial biofilms in medicine: clinical implications

Since the concept emerged, biofilms have been recognized as an especially important pathogenic factor in human infections (Hall-Stoodley and Stoodley, 2005). Although acute infections are mainly caused by planktonic organisms, the pathogenesis of chronic diseases seems to be strongly associated with biofilm formation. Moreover, the growing importance of biomaterials, such as catheters or prostheses, in modern medicine has contributed to the significance of biofilms and their management in human disease (Patino et al., 2008; Qvist et al., 2014, 2015).

The clinical relevance of mycobacterial biofilms can be analyzed in two groups: biofilms in NTM disease and biofilms in tuberculosis. These two groups will be analyzed below.

### Non-tuberculous mycobacterial disease

The spectrum of diseases caused by NTM is wide, but almost all of them share a common characteristic: they are usually chronic.

One of the most common syndromes caused by NTM is respiratory disease (Wallace et al., 1983; Griffith et al., 2007; Esteban et al., 2012), caused either by slowly growing mycobacteria, such as *M. avium* complex, or rapidly growing species, such as *M. abscessus*. Respiratory disease usually affects patients with predisposing conditions, such as old tuberculosis scars, silicosis, bullae, and other lung cavities where NTM can develop a biofilm that first colonizes the host, and later causes invasive disease. This syndrome was first recognized several decades ago (Wolinsky, 1979, 1992), and has always been difficult to manage. In recent times, new populations of patients especially susceptible to these pathogens have been identified. These include patients with chronic bronchiectasis and cystic fibrosis, conditions which have been recently reported as predisposing factors for NTM infections (Benwill and Wallace, 2014; Qvist et al., 2014; Floto et al., 2016; Hoiby et al., 2017). The association between these infections and biofilm has been documented (Qvist et al., 2015). Mycobacterial biofilms have recently been identified in histological samples from lung cavitary disease (Fennelly et al., 2016). Experimental data also show the importance of biofilm development in the ability of *M. avium* to invade bronchial epithelial cells (Yamazaki et al., 2006).

Another important biofilm-related group of infections, and the most typical, are those associated with biomaterial. NTM have been reported as the cause of different syndromes, the most frequently isolated organisms being RGM. These organisms behave as opportunistic pathogens and have been described in many different device-related infections. Among these, catheter-related bacteremia has been reported in many different populations, including cancer patients (El Helou et al., 2013b). Diagnosis is difficult, but in some cases these organisms can grow in conventional blood culture bottles. Catheter removal is mandatory for the management of these infections in all cases (El Helou et al., 2013a,b). Another catheter-related disease is peritonitis in continuous ambulatory peritoneal dialysis (CAPD) patients. This syndrome has been described since the early days of CAPD use, and also requires catheter removal if cure is to be achieved (Hakim et al., 1993).

Other devices that can be infected with NTM are prosthetic joints. The most common causative agents are RGM (Eid et al., 2007), but slowly growing mycobacteria have also been isolated (Gupta and Clauss, 2009), albeit rarely (Benito et al., 2016). Although RGM can grow in common bacteriology media, slowly growing organisms usually require specific mycobacterial culture methods, so a high degree of suspicion is necessary before such diagnostic procedures are undertaken. Other biomaterial-related diseases include abdominal mesh infection (Celdran et al., 2007), pacemaker infection (Al-Ghamdi et al., 2016), prosthetic valve endocarditis (Bouchiat et al., 2015), mammoplasty infection, transplant-related keratitis, and others (Brown-Elliott and Wallace, 2002). Almost all of these syndromes require implant removal, since antibiotic therapy alone is incapable of eliminating the sessile organisms.

### *Mycobacterium tuberculosis* disease

Arguably one of the most interesting findings in recent years in the field of mycobacterial biofilm research is the discovery that *M. tuberculosis* can develop a biofilm. The first reports came from cases of tuberculosis infection associated with clinical biomaterial, prosthetic joints in particular (Tokumoto et al., 1995; Spinner et al., 1996; Berbari et al., 1998; Ha et al., 2005). Clinical observations led to the conclusion that biomaterial removal was essential to manage these infections, even if the *M. tuberculosis* strain was susceptible *in vitro* to the antibiotics used. The difficulty in treating these infections could be due to the fact that biofilm is a well-established mechanism of antibiotic resistance. Further studies have indeed demonstrated that *M. tuberculosis* can develop a biofilm *in vitro*, opening a new line of research in the pathogenesis of this disease (Ojha et al., 2008). Since then, several studies have determined the importance of different molecules, such as mycolic acids or DNA, in the development of *M. tuberculosis* biofilms, and the different regulatory mechanisms involved in this process have been revealed (Nayak, 2015; Ojha et al., 2015). However, the role of biofilms in the pathogenesis of tuberculosis remains unclear. This pathogenic process is complex, and involves, most importantly, intracellular survival and host defense evasion mechanisms. It has been suggested that the importance of biofilms in this disease is due to their participation in the process of casseous necrosis and cavitation formation in lung tissue, a site in which *M. tuberculosis* could form a biofilm (Kulka et al., 2012; Basaraba and Ojha, 2017). Further experiments have shown a decrease in the activity of antituberculous drugs against tuberculosis biofilms (Ojha et al., 2008; Islam et al., 2012). These discoveries prompted interest in biofilm-forming mechanisms as a potential target for new therapies against tuberculosis. Nevertheless, the clinical implications of these *in vitro* discoveries remain unresolved, and future research will probably furnish us with a new view of tuberculosis as a biofilm-related disease, to add to its other pathogenic factors.

## Mycobacterial biofilms in medicine: therapeutic implications

Biofilm development is an important factor in antimicrobial resistance. It affords many bacterial species protection against antibiotics normally active against the same bacteria in the planktonic state (Hoyle and Costerton, 1991; Fux et al., 2005; Ciofu et al., 2017). Different mechanisms have been implicated in this resistance (permeability, metabolic states, activation of resistance genes, persister cells) (Anderson and O'Toole, 2008; Lewis, 2008; Kester and Fortune, 2014). Resistance to antibiotics, disinfectants, and germicides by biofilm-forming microorganisms may lead to treatment failure, and clinical experience has demonstrated that biofilms have to be physically eradicated to resolve the infection (Hall-Stoodley et al., 2012). Several studies have found mycobacterial biofilms resistant *in vitro* to disinfectants or antibiotics, including amikacin and clarithromycin. For example, even when minimal inhibitory concentrations (MIC) indicated that an *M. abscessus* isolate was susceptible to amikacin and clarithromycin, these drugs were only minimally active in biofilms at the highest concentrations tested (Greendyke and Byrd, 2008; Ortiz-Perez et al., 2011). Muñoz-Egea et al. found differences between the MIC and minimum biofilm eradication concentration (MBEC) in 4 species of RGM, ranging between <100-fold in the case of *Mycobacterium mucogenicum* exposed to ciprofloxacin, and >100,000-fold in the case of *M. abscessus* and *Mycobacterium peregrinum* exposed to clarithromycin (Muñoz-Egea et al., 2015); ciprofloxacin was the most active antibiotic against these biofilms, compared with clarithromycin or amikacin. Further studies have shown the effect of antibiotic therapy in different stages of biofilm development (Muñoz-Egea et al., 2015, 2016b). In these studies, treatment of the biofilm was more effective when antibiotics are added in the early stage of biofilm development, probably because the phenotype of the cells is not fully adapted to biofilm growth.

In an attempt to evaluate mechanisms for these resistance patterns, Ortiz-Pérez et al. examined the permeability of mycobacterial biofilm to different antimicrobials. These authors studied several clinical and laboratory strains and found that antimicrobial permeability features were not species-dependent or related to drug resistance of the biofilm (Ortiz-Perez et al., 2011). Greendyke and Byrd demonstrated that in *M. abscessus* the metabolic state is essential for the development of resistance (Greendyke and Byrd, 2008). Other mechanisms, including activation of resistance genes [such as inducible methylases, found in many species of mycobacteria (Esteban et al., 2009)] have been hypothesized, but remain unproven.

The implications of biofilm development in the resistance of *M. tuberculosis* to antimicrobials have been demonstrated (Ojha et al., 2008), although it is not clear how these findings will apply to the treatment of tuberculosis.

New strategies to improve treatment efficacy and outcomes in patients with infections caused by these organisms have been studied. Differences in biofilm development and structure between species may require different approaches, depending on the mycobacteria involved. Muñoz-Egea et al. studied the effect of N-acetylcysteine (NAC) and Tween 80 (two potential antibiofilm molecules), alone and combined with antibiotics, against non-pigmented RGM (NPRGM) biofilms. Tween 80 alters the structural integrity of the membrane, lipids, and proteins (Teixeira et al., 2007), while NAC acts on the polysaccharide matrix of the biofilm, breaking disulfide bridges that link the polysaccharide fibers (Olofsson et al., 2003). Due to the high lipid content of the mycobacterial cell wall and the significant presence of lipids in the extracellular matrix, Tween 80 is more active against mycobacterial biofilm than NAC (Muñoz-Egea et al., 2016b). An increase in antibacterial activity was observed when NAC and Tween 80 were combined with ciprofloxacin, clarithromycin, and amikacin. The ultrastructure of biofilms in *M. fortuitum, M. chelonae, M. abscessus*, and *M. smegmatis* is also affected by ciprofloxacin, clarithromycin, and amikacin combined with antibiofilm agents. In fact, the percentage of dead bacteria is higher with a combination of antibiotics and antibiofilm agents than with antibiotics only (Muñoz-Egea et al., 2016b). This synergistic effect is potentially useful in the prophylaxis or treatment of infections associated with mycobacterial biofilms.

A new antibiofilm strategy is currently under investigation. *Methylobacterium* sp. is a Gram-negative *Alphaproteobacteria* usually found in water distribution systems, that, when isolated in biofilms, has been linked with a lower presence of *M. avium* (Falkinham et al., 2016). In a subsequent study with *M. abscessus*, Muñoz-Egea et al. showed that *Methylobacterium* sp. can inhibit *M. abscessus* biofilm formation, affecting both thickness and surface area. This study also demonstrated that *Methylobacterium* sp. do not have to be live to inhibit a preformed biofilm of *M. abscessus:* the addition of a crude extract of autoclaved *Methylobacterium* sp. inhibited biofilm development (Muñoz-Egea et al., 2016a). This new approach could lead in the future to the discovery of new antibiofilm agents with specific activity against some species of mycobacteria. This approach is potentially of great interest in the treatment of the numerous infections in which biofilm cannot be surgically removed from the patient.

## Conclusions

Mycobacterial biofilms constitute a specific field in this line of research and present unique characteristics. The importance of these structures in environmental and clinical settings is beyond doubt. In the clinic, understanding of biofilms is essential for the proper management of many NTM diseases, especially those associated with biomaterials, because these biofilms render the mycobacteria resistant to commonly used antibiotic treatments. New strategies in the management of this disease are called for, especially when infected tissue cannot be removed. The recent discovery of tuberculosis biofilms provides a new perspective to this extremely important disease, and further study of *M. tuberculosis* biofilms could alter treatment strategies for this entity in the years to come.

## Author contributions

Both authors perform the review of the articles and write the manuscript in the same proportion. JE: supervised all the work and perform the final corrections.

### Conflict of interest statement

The handling Editor declared a past collaboration with one of the authors JE. The other authors declare that the research was conducted in the absence of any commercial or financial relationships that could be construed as a potential conflict of interest.
